# Is ChatGPT an Effective Tool for Providing Dietary Advice?

**DOI:** 10.3390/nu16040469

**Published:** 2024-02-06

**Authors:** Valentina Ponzo, Ilaria Goitre, Enrica Favaro, Fabio Dario Merlo, Maria Vittoria Mancino, Sergio Riso, Simona Bo

**Affiliations:** 1 Department of Medical Sciences, University of Torino, 10126 Torino, Italy; valentina.ponzo@unito.it (V.P.); ilaria.goitre@unito.it (I.G.); enrica.favaro@unito.it (E.F.); 2 Dietetic and Clinical Nutrition Unit, Città della Salute e della Scienza Hospital of Torino, 10126 Torino, Italy; fmerlo2@cittadellasalute.to.it (F.D.M.); mmancino@cittadellasalute.to.it (M.V.M.); 3 Dietetic and Clinical Nutrition Unit, Azienda Ospedaliero-Universitaria Maggiore della Carità of Novara, 28100 Novara, Italy; sergio.riso@maggioreosp.novara.it

**Keywords:** ChatGPT, dietary advice, guidelines, non-communicable diseases (NCDs)

## Abstract

The chatbot Chat Generative Pretrained Transformer (ChatGPT) is becoming increasingly popular among patients for searching health-related information. Prior studies have raised concerns regarding accuracy in offering nutritional advice. We investigated in November 2023 ChatGPT’s potential as a tool for providing nutritional guidance in relation to different non-communicable diseases (NCDs). First, the dietary advice given by ChatGPT (version 3.5) for various NCDs was compared with guidelines; then, the chatbot’s capacity to manage a complex case with several diseases was investigated. A panel of nutrition experts assessed ChatGPT’s responses. Overall, ChatGPT offered clear advice, with appropriateness of responses ranging from 55.5% (sarcopenia) to 73.3% (NAFLD). Only two recommendations (one for obesity, one for non-alcoholic-fatty-liver disease) contradicted guidelines. A single suggestion for T2DM was found to be “unsupported”, while many recommendations for various NCDs were deemed to be “not fully matched” to the guidelines despite not directly contradicting them. However, when the chatbot handled overlapping conditions, limitations emerged, resulting in some contradictory or inappropriate advice. In conclusion, although ChatGPT exhibited a reasonable accuracy in providing general dietary advice for NCDs, its efficacy decreased in complex situations necessitating customized strategies; therefore, the chatbot is currently unable to replace a healthcare professional’s consultation.

## 1. Introduction

Noncommunicable diseases (NCDs) constitute the foremost global cause of mortality, representing 74% of deaths worldwide [1]. Among them, cardiovascular diseases (CVD) emerge as the primary contributors to this alarming statistic, being responsible for an estimated 18.6 million deaths [2]. According to global estimates from the 2019 Global Burden of Diseases (GBD) study, there were approximately 43.8 million cases of type 2 diabetes (T2DM), 18.5 million cases of hypertension, and 1.2 billion cases of non-alcoholic fatty liver disease (NAFLD) worldwide [3]. The overall prevalence of obesity nearly tripled from 1975 to 2016 [4], reaching 14.0% in 2019 [5]. Several research studies have consistently highlighted the relevant impact of dietary and lifestyle factors on the onset and progression of most NCDs [6].

Internet searches for health-related information are a constantly growing phenomenon [7]. Chat Generative Pretrained Transformer (ChatGPT) is one of the most widely used chatbots developed by OpenAI, with an advanced language employing sophisticated machine learning algorithms to generate responses to text-based queries, mimicking human-like conversation [8]. The chatbot can understand the context and provide accurate and coherent responses [9]. Owing to its personalized and interactive approach and the immediate, free, easily accessible information across different platforms and devices, it is emerging as an efficient and accessible resource for individuals seeking online medical advice [10]. An increasing interest is currently observed among healthcare professionals in the use of this innovative tool to enhance patient care, diagnosis, and treatment [11]. A growing application of artificial intelligence (AI) is noted in the domain of patient education and support. Chatbots demonstrate the capacity to deliver real-time, personalized, and interactive education and support to patients, thereby improving overall patient engagement and outcomes [11,12]. Limited literature is, however, available at present on the use of ChatGPT as a tool for improving nutrition knowledge in patients with different chronic non-communicable diseases (NCDs) [13,14]. A recent study exploring the potential of ChatGPT in delivering nutritional information concluded that this tool cannot replace the expertise of a registered dietitian, particularly in addressing complex medical conditions [13]. The authors identified limitations such as incorrect responses, absence of hands-on demonstration, inability for physical examination, incapacity to proactively engage patients, and the lack of verbal and non-verbal cues [13]. The effectiveness of ChatGPT in addressing common nutrition inquiries was also explored, and its responses were compared with those from human experts. The results indicated that the chatbot performed on par with human dietitians in delivering accurate answers [15]. The ability of ChatGPT to generate appropriate and personalized meal plans for patients with NCDs demonstrated promising results, but the given recommendations exhibited around 20% mean caloric differences with respect to the target energy intake value [16]. Finally, concerns about the safety and accuracy of dietary plans generated by chatbots have been raised. A study assessing the abilities of ChatGPT to define the ideal diet and meal plans for a hypothetical patient with type 2 diabetes mellitus and a patient undergoing hemodialysis reported that the answers of the chatbot were clear and relatively accurate, but the meal plan for the patient undergoing hemodialysis included inappropriate foods [17]. Concern about the accuracy of diets provided by ChatGPT was raised by a study assessing 56 diets created by ChatGPT for hypothetical individuals with food allergies due to the findings of inaccuracies, with errors involving portions or calories of food, meals, or whole diets [18].

To the best of our knowledge, no previous study has rigorously evaluated whether the dietary advice provided by ChatGPT is appropriate in comparison with recommendations from international guidelines. The objective of the current study was therefore to compare the nutritional information supplied by ChatGPT regarding different NCDs requiring dietary advice with the nutritional recommendations from international guidelines. Additionally, we aimed to assess whether the chatbot could serve as a substitute for dietitians in delivering personalized dietary advice in patients with multiple NCDs.

## 2. Materials and Methods

### 2.1. ChatGPT Version and Selection of Medical Condition

The study was conducted in November 2023 using the ChatGPT default model (OpenAI, San Francisco, CA, USA, version 3.5).

Several medical conditions requiring specific dietary treatments were included in the study:

- Dyslipidemia (hypercholesterolemia and hypertriglyceridemia);
- Arterial hypertension;
- Type 2 diabetes mellitus (T2DM);
- Obesity;
- Non-alcoholic fatty liver disease (NAFLD);
- Chronic kidney disease (CKD);
- Sarcopenia.

Initially, dietary advice for metabolic-dysfunction-associated steatotic liver disease (MASLD) was sought from ChatGPT. However, as the chatbot’s knowledge is limited to updates until January 2022, it was unable to provide a response. Consequently, the inquiry was redirected towards NAFLD.

### 2.2. Experiment 1

#### 2.2.1. Prompt Selection

The appropriateness of dietary advice given by ChatGPT was assessed by formulating a set of prompts for the medical conditions listened above. The questions were formulated by a panel of experts composed by medical doctors and registered dietitians using language and sentence structure intended to replicate how patients might inquire with a healthcare professional. Three types of prompts were tested for each medical condition:

- “Could you provide guidance on planning an optimal diet to manage [disease]?”
- “What are the dietary recommendations for [disease]?”
- “I have [disease], what should I eat?”

All prompts were conducted on 3 November 2023, and each prompt conversation with ChatGPT was performed in English using separate chat sessions for each prompt. ChatGPT can generate different responses to identical prompts depending on the context and conversation history. Each question was therefore posed three times, and each of the three inquiries was conducted within a new chat session to prevent any potential bias related to the model’s memory. The responses exhibited slight variations depending on the prompt, although the listed recommendations remained substantially consistent, with few minimal variations. Consequently, the most comprehensive answer, i.e., the one that presented the highest number of information in alignment with the guidelines, was considered.

#### 2.2.2. Analysis of Guideline Agreement

The appropriateness of each response from ChatGPT was compared to guidelines recommendations (Table 1). The prompt with the most comprehensive answer for each question is reported in bold in the table.

ChatGPT answers were independently assessed and categorized by two healthcare professionals (two dietitians) who were blinded to each other’s evaluations of ChatGPT responses. The first dietitian has over 20 years of expertise in the field of clinical nutrition, malnutrition, sarcopenia, diabetes, and kidney disease. The second dietitian holds a Ph.D. in medical pathophysiology and has 12 years of experience in NCDs (obesity, diabetes, cardiovascular diseases, and NAFLD). Controversies were resolved by a third reviewer (a medical doctor). ChatGPT advice was designated as “appropriate” if the content was in accordance with the guidelines, “inappropriate” if contradicted the guidelines, “not supported” if did not find confirmation in the guidelines, and “not fully matched” if it did not fully fulfill the recommendations of the guidelines. Meanwhile, each ChatGPT advice that was not specifically focused but rather promoting overall a healthy diet was classified as “general advice”. In addition, any dietary guideline recommendation that was not present in the ChatGPT response was labeled as “missing”. 

### 2.3. Experiment 2

The chatbot’s ability to substitute a consultation with a dietitian by managing complex cases was investigated.

#### 2.3.1. Prompts Selection

To evaluate ChatGPT’s ability to incorporate multiple dietary recommendations, a more intricate scenario involving the coexistence of multiple conditions (type 2 diabetes, obesity, and chronic kidney disease) was presented to the chatbot.

#### 2.3.2. Measures

ChatGPT’s responses were evaluated by a panel of experts including two dietitians and one medical doctor. 

### 2.4. Ethical Considerations

Ethical committee approval for this study was not deemed necessary, as it did not enroll human participants or animals.

## 3. Results

### 3.1. Experiment 1

Comprehensive responses from ChatGPT to the prompts and the comparison with guideline recommendations are presented in Supplementary Table S1.

There were very few instances of dispute between the dieticians assessing ChatGPT’s answers; specifically, four cases (4.4%) required the reviewer’s intervention to be resolved.

Overall, ChatGPT’s advice was accurate, with appropriateness rates ranging from 55.5% for sarcopenia up to 73.3% for NAFLD (Table 2). If we consider all the correct recommendations, including both “appropriate” and “general advice”, the overall accuracy of nutritional recommendations reached 100% for sarcopenia. Few recommendations were found to be in contradiction with the guidelines: one for obesity and one for NAFLD (Table 2). A discrepancy emerged for obesity management because ChatGPT advocated for stabilizing blood sugar levels through regular meals and snacks, while the guidelines of the European Association for the Study of Obesity (EASO) emphasized the avoidance of snacking between meals [23]. Regarding NAFLD, ChatGPT reported the potential benefits from supplements such as vitamin E, omega-3 fatty acids, and antioxidants under medical supervision, while the European Society for Clinical Nutrition and Metabolism (ESPEN) cautiously asserted that, pending further data on efficacy, omega-3 fatty acids and antioxidants (e.g., vitamin C, resveratrol, anthocyanin, and bayberries) cannot be endorsed for treating NAFLD [28].

One recommendation relative to T2DM was identified as unsupported by the guidelines, although not explicitly conflicting (Table S2). ChatGPT advised spreading meals throughout the day, suggesting smaller, balanced meals and snacks as an alternative to three large meals. This advice did not directly contradict established guidelines but was not addressed by existing T2DM guidelines.

Several discrepancies classified as “not fully matched” were identified between ChatGPT’s dietary recommendations and guidelines. ChatGPT emphasized monitoring portion sizes and incorporating low-glycemic-index foods for hypertriglyceridemia, while guidelines recommended carbohydrate reduction and addressing excessive body weight as key strategies. The chatbot advocated for controlling portion sizes to prevent overeating and subsequent blood sugar spikes in T2DM, suggesting the use of smaller plates, bowls, and utensils. Guidelines recommended evidence-based treatments to support individuals with diabetes and weight excess, with a recommended minimum 5% weight loss. Discrepancies labeled as “not fully matched” for CKD pertained to protein intake. ChatGPT suggested a nonspecific limitation in protein intake depending on the stage of CKD, while guidelines offered detailed recommendations regarding protein allowances tailored to the specific CKD stage. 

In addition to condition-specific recommendations, ChatGPT provided generic health advice for many different diseases. Suggestions such as “staying hydrated” (hypertriglyceridemia, T2DM, CKD, obesity, arterial hypertension, NAFLD, and sarcopenia), “incorporating lean proteins”, and “avoiding processed foods” (T2DM and arterial hypertension) were repeatedly reported and aspects such as appetite management and overall well-being addressed several times. Overall, ChatGPT advice was generic, providing practical examples of foods to be included in the diet, with the latter information not often reported in guidelines.

### 3.2. Experiment 2

The conversation with ChatGPT relative to a hypothetical patient with T2DM, obesity, and CKD is reported in Table 3. A few suggestions were conflicting or inappropriate. ChatGPT suggested prioritizing lean proteins to support muscle health but subsequently advised to limit overall protein intake. Information regarding fluid intake according to the stage of CKD was not reported. Additionally, key recommendations related to phosphorus and potassium intakes were omitted as well as guidance on adjusting dietary intakes according to the CKD stage. 

In general, ChatGPT’s response appeared to be generic. When the CKD stage was inputted into the prompt to verify if the answer would be more targeted, ChatGPT segmented the recommendations for the three conditions without integrating them. The importance of consulting a registered dietitian for a meal plan tailored to specific requirements was emphasized repeatedly.

## 4. Discussion

Several points of agreement and few divergences were identified by comparing ChatGPT’s dietary advice with the recommendations provided by international guidelines for different NCDs. The answers of the chatbot were generally clear and in line with the recommendations provided by the guidelines. Additionally, ChatGPT provided practical examples specifying which foods to include or exclude from the diet. However, the answers of the chatbot were partially complete, with a few guideline recommendations missing, above all for T2DM, obesity, dyslipidemia, NAFLD, and CKD. Additionally, ChatGPT was unable to offer suitable guidance when numerous conditions coexisted in the same patient. 

Our results were partially consistent with previous studies. The ability of ChatGPT to address eight commonly asked nutritional questions (such as “Can I use intermittent fasting to lose weight more quickly?” or “Are carbohydrates bad?”) was compared to the answers of registered dietitians and experts in the corresponding fields by evaluating the “scientific correctness”, “comprehensibility”, and “actionability”, grading on a scale from 0 to 10 [15]. According to the study’s findings, ChatGPT consistently outperformed human dietitians in all three criteria across the majority of questions, and authors concluded that the chatbot demonstrated an at least comparable if not superior proficiency in responding to common nutritional inquiries compared to human dietitians and/or experts in the field [15]. In this study, only generic questions were addressed; however, exploring more complex or specific queries, such as those related to specific food categories or providing more detailed patient information, would have thoroughly tested the chatbot’s ability to deliver accurately personalized and up-to-date responses. Additionally, emotional aspects and the ability to customize responses based on individual needs are critical aspects of the interaction between a patient and a care professional, and ChatGPT lacks the capacity and sensitivity to adapt to the unique needs of each individual.

The ability of ChatGPT to offer advice for T2DM patients was reported to be adequate with clear answers, generally aligned with the information provided by the American Diabetes Association (ADA) [17]. The chatbot’s ability to create meal plans for hypothetical patients with T2DM or those undergoing to hemodialysis was also assessed [17]. Limitations were found in the tailoring of the meal plans to the specific pathological condition since foods included in the menus were suboptimal for hemodialysis patients, and the meal plans exhibited repetition, resembling those generated for T2DM patients [17]. Similar limitations were identified by other researchers investigating food allergies [18]. The inclusion of inappropriate foods in dietary plans by ChatGPT was found (e.g., almond milk was included in a nut-free meal plan) [18]. 

A recent study explored the capability of providing personalized diets for hypothetical patients with obesity, CVD, and T2DM in terms of energy intake, nutrient accuracy, and meal variability [16]. The meal plans generated by ChatGPT were not monotonous, but the recommended daily caloric intakes deviated from the target energy intake, with differences up to 20% [16]. Notably, when the target’s energy intake was specifically requested in the prompt, there was a significant reduction in caloric deviations from the optimal energy intake. The authors observed that with ChatGPT version 3.5, the deviation decreased from 19.6% to 17.3%, while with version 4, it decreased from 27.7% to 3.4% [10]. These results suggest that ChatGPT-based recommendations can benefit significantly from the inclusion of additional user information resulting in the generation of more targeted meal plans [16]. 

Our findings indicated that the chatbot had limitations when integrating various recommendations due to the occurrence of multiple health issues. It was unable to combine different recommendations, which resulted in contradictory or inappropriate advice that could have confused users. When comparing our findings with those of earlier research, ChatGPT was able to provide broad nutritional recommendations for different health issues, but it did not consistently exhibit accuracy in delivering tailored dietary advice or plans. It became clear that chatbots cannot replace the personalized advice provided by a healthcare professional. However, ChatGPT never claimed to be a substitute. Every response made clear that ChatGPT is a research chatbot and should not be used for medical purposes. Additionally, a closing note was included emphasizing the importance of consulting a healthcare provider or a registered dietitian in case of underlying medical conditions. Finally, another limitation that emerged from ChatGPT’s responses was the absence of warnings or notifications regarding food allergies for the recommended foods. 

Notwithstanding these concerns, ChatGPT is a promising tool that, with further development, may prove helpful for nutrition advice. Indeed, there are a lot of potential benefits for patients using chatbots, but there are also some drawbacks and hazards. ChatGPT is a platform that makes it easy and simple for people to obtain nutritional advice and assistance whenever they want, regardless of where they are, thus improving their access to education and support [12]. Users can access it on a variety of devices, including PCs, tablets, and smartphones, providing them flexibility in how they receive information. This could be especially helpful in addressing access to healthcare information for people living in underserved or rural locations [12]. ChatGPT’s round-the-clock accessibility guarantees that patients may receive help and assistance whenever they need it, which lessens reliance on the sometimes-limited availability of healthcare providers [33]. It also avoids the long waitlists for professional consultations and is free of cost.

However, potential risks and limitations in using ChatGPT for medical advice have been raised, limiting its potential application in healthcare to date. First, the inappropriate or wrong nutritional advice provided by the ChatGPT could lead to negative health outcomes for patients. Moreover, a limitation is the lack of current updates on ChatGPT, with the last update dating back over a year ago. Therefore, it may offer information that is not based on the latest available evidence. Furthermore, the use of ChatGPT in nutritional education might be limited by the patient’s digital literacy [12]. While younger individuals may readily embrace technology, older patients may not be as willing to do so and might encounter difficulties understanding and using the platform. Finally, ethical concerns arise in the utilization of ChatGPT in this context since patients may share sensitive health information with the ChatGPT system. Rigorous measures must be implemented to safeguard the privacy and confidentiality of patient data, aiming to prevent any unauthorized disclosure of sensitive health information [10]. Addressing ethical considerations is of utmost importance to guarantee the safety and efficacy of the ChatGPT system [12].

### Limitations of the Study

We utilized ChatGPT version 3.5; however, there are now more recent versions and, most importantly, other chatbots accessible. It is important to acknowledge that ChatGPT’s responses to identical prompts are subject to change over time due to the unpredictable nature of the underlying model and the ongoing refinements in ChatGPT’s performance [8]. As a result, figuring out the precise information a patient might receive becomes difficult, particularly if the prompts include extra parameters such as gender, age, food preferences, or other personal information. Furthermore, it is possible that the patient-focused questions we asked ChatGPT did not cover all the possible queries a patient would have for a dietician. Lastly, we only consider the output produced by ChatGPT, even though users usually interact with ChatGPT via a sequence of prompts rather than a single conversation. In fact, ChatGPT can engage in numerous rounds of dialogue, which enables users to seek clarification, in contrast to traditional web searches.

## 5. Conclusions

ChatGPT proved to be fairly accurate in providing responses related to nutritional advice for various NCDs. However, it exhibited limitations in handling more complex cases involving the coexistence of multiple health conditions. Consequently, even while it shows potential utility, to date, it cannot replace the advice of a health care professional with expertise in nutrition.

## Figures and Tables

**Table 1:** Set of prompts and the clinical guidelines used to compare answers for each question topic.

Question Topic	Set of Prompts	Clinical Guideline
Dyslipidemia	– Could you provide guidance on planning an optimal diet to manage hypercholesterolemia? – What are the dietary recommendations for hypercholesterolemia? – **I have hypercholesterolemia, what should I eat?** ----------------------------------- – **Could you provide guidance on planning an optimal diet to manage hypertriglyceridemia?** – What are the dietary recommendations for hypertriglyceridemia? – I have hypertriglyceridemia, what should I eat?	European Society of Cardiology (ESC) and the European Atherosclerosis Society (EAS)-Guidelines for the management of dyslipidaemias (2019) [19] American Heart Association (AHA) and American College of Cardiology (ACC)-Guideline on the Management of Blood Cholesterol: A Report of the American College of Cardiology/American Heart Association Task Force on Clinical Practice Guidelines (2018) [20]
Arterial hypertension	– Could you provide guidance on planning an optimal diet to manage hypertension? – **What are the dietary recommendations for hypertension?** – I have hypertriglyceridemia, what should I eat?	The European Society of Hypertension (ESH)-Guidelines for the management of arterial hypertension (2023) [21]. International Society of Hypertension (ISH)-Global Hypertension Practice Guidelines (2020) [22].
Obesity	– Could you provide guidance on planning an optimal diet to manage obesity? – **What are the dietary recommendations for obesity?** – I have obesity, what should I eat?	European Association for the Study of Obesity/EASO)-European Guidelines for Obesity Management in Adults (2015) [23]. Canadian Adult Obesity Clinical Practice Guidelines: Medical Nutrition Therapy (updated 2022) [24]
Type 2 Diabetes Mellitus (T2DM)	– **Could you provide guidance on planning an optimal diet to manage type 2 diabetes mellitus?** – What are the dietary recommendations for type diabetes mellitus? – I have type 2 diabetes mellitus, what should I eat?	American Diabetes Association (ADA)—Standard of care (2023) [25] The Diabetes and Nutrition Study Group (DNSG) of the European Association for the Study of Diabetes (EASD). Evidence-based European recommendations for the dietary management of diabetes (2023) [26]
Non-alcoholic fatty liver disease (NAFLD)	– Could you provide guidance on planning an optimal diet to manage NAFLD? – **What are the dietary recommendations for NAFLD?** – I have NAFLD, what should I eat?	American Association for the study for Liver Diseases (AASLD)-Practice Guidance on the clinical assessment and management of nonalcoholic fatty liver disease (2023) [27] ESPEN practical guideline: Clinical nutrition in liver disease (2020) [28]
Chronic kidney disease (CKD)	– **Could you provide guidance on planning an optimal diet to manage chronic kidney disease?** – What are the dietary recommendations for chronic kidney disease? – I have chronic kidney disease, what should I eat?	KDOQI-Clinical practice guideline for nutrition in CKD: 2020 update [29] Clinical practice guideline for the evaluation and management of chronic kidney disease-KDIGO 2023 [30]
Sarcopenia	– Could you provide guidance on planning an optimal diet to manage sarcopenia? – **What are dietary recommendations for sarcopenia?** – I have sarcopenia, what should I eat?	Protein intake and exercise for optimal muscle function with aging: Recommendations from the ESPEN Expert Group (2014) [31] International Clinical Practice Guidelines for Sarcopenia (ICFSR): Screening, Diagnosis and Management (2018) [32]

The most comprehensive answer for each question is reported in bold.

**Table 2:** Classification of ChatGPT responses in comparison to guideline recommendations.

Topic	Appropriate	Not Fully Matched	General Advice	Not Supported	Inappropriate	Missing
Hypercholesterolemia Hypertriglyceridemia	15 (71.4%)	2 (9.5%)	4 (19.0%)	0	0	2
Arterial hypertension	8 (66.7%)	1 (8.3%)	3 (25.0%)	0	0	1
Obesity	7 (70.0%)	0	2 (20.0%)	0	1 (10.0%)	2
Type 2 diabetes mellitus	10 (71.4%)	1 (7.1%)	2 (14.3%)	1 (7.1%)	0	3
NAFLD	11 (73.3%)	1 (6.7%)	2 (13.3%)	0	1 (6.7%)	2
Chronic kidney disease	7 (70.0%)	1 (10.0%)	2 (20.0%)	0	0	2
Sarcopenia	5 (55.6%)	0	4 (44.4%)	0	0	0

**Table 3:** Conversation with ChatGPT.

Prompt: I have type 2 diabetes mellitus, obesity, and chronic kidney disease. Can you give me nutritional advice?
ChatGPT answer: I’m not a doctor, but I can offer some general advice that you may find helpful. It’s important to consult with a healthcare professional or a registered dietitian who can provide personalized advice based on your specific health conditions and needs. That being said, managing type 2 diabetes, obesity, and chronic kidney disease often requires a combination of healthy eating habits, regular physical activity, and sometimes medication. Here are some general dietary tips that may be beneficial, but please remember to consult with your healthcare team before making any significant changes: Control Portion Sizes: Be mindful of portion sizes to help manage blood sugar levels and support weight management. Smaller, balanced meals can help regulate blood sugar levels throughout the day. Choose Complex Carbohydrates: Opt for complex carbohydrates like whole grains, legumes, and vegetables, as they have a slower impact on blood sugar compared to refined carbohydrates. Limit Added Sugars: Minimize your intake of foods and beverages with added sugars, as they can lead to rapid spikes in blood sugar levels. Prioritize Lean Proteins: Include lean protein sources like poultry, fish, tofu, legumes, and low-fat dairy products. These can help with satiety and support muscle health. Healthy Fats: Choose heart-healthy fats like avocados, nuts, seeds, and olive oil in moderation. Avoid trans fats and limit saturated fats. Monitor Sodium Intake: If you have chronic kidney disease, it’s important to monitor your sodium intake to help manage blood pressure and kidney function. Moderate Protein Intake: Depending on the severity of your chronic kidney disease, you may need to adjust your protein intake. A registered dietitian can help determine the appropriate level for you. Stay Hydrated: Drink an adequate amount of water throughout the day. Proper hydration is important for kidney function. Limit Processed Foods: Processed foods are often high in sodium, added sugars, and unhealthy fats. Try to choose whole, unprocessed foods whenever possible. Fiber-Rich Foods: Include plenty of fiber from fruits, vegetables, and whole grains. Fiber can help regulate blood sugar levels and support digestive health. Regular Monitoring: Monitor your blood sugar levels regularly and keep track of what you eat to identify patterns and make necessary adjustments. Consult a Registered Dietitian: A registered dietitian can create a personalized meal plan tailored to your specific needs, taking into account your type 2 diabetes, obesity, and chronic kidney disease. Remember, individual nutritional needs can vary, so it’s crucial to work with a healthcare professional who can provide personalized guidance based on your unique situation. They can also help you establish realistic goals and monitor your progress over time.

## Data Availability

Data are contained within the article and Supplementary Materials.

## Supplementary Materials

The following supporting information can be downloaded at: https://www.mdpi.com/article/10.3390/nu16040469/s1, Table S1: Comparison between advice provided by ChatGPT and guideline recommendations. Table S2: Inappropriate, unsupported, and missing advice from ChatGPT responses.
