# Supplementary material for: Is ChatGPT an Effective Tool for Providing Dietary Advice?
Source: Nutrients. 2024 Feb 6;16(4):469. doi: 10.3390/nu16040469 (PMC10892804; doi:10.3390/nu16040469)
Supplement: Supplementary file 1 [file nutrients-16-00469-s001.zip › Table S1.pdf]

Table S1. Comparison between advice provided by chatGPT and guideline recommendations.

Color legend: Appropriate= green; Not fully matched = grey; General advice= blue; Not supported= yellow; Inappropriate= red; Missing= orange

Chat GPT dietary advice	Guideline dietary recommendations
Hypercholesterolemia	
I'm not a doctor, but I can offer some general dietary suggestions for people with hypercholesterolemia. It's important to consult with a healthcare professional or a registered dietitian for personalized advice and a treatment plan tailored to your specific needs.<br>Hypercholesterolemia refers to high levels of cholesterol in the blood. To manage this condition, it's typically recommended to focus on a heart-healthy diet that aims to lower levels of LDL (low-density lipoprotein) cholesterol, often referred to as "bad" cholesterol. Here are some dietary tips that may help:<br>1. Limit Saturated and Trans Fats:<br>• Reduce intake of foods high in saturated fats like fatty meats, full-fat dairy products, and certain oils (e.g., coconut oil, palm oil).<br>• Avoid trans fats, which are often found in processed and fried foods. Check food labels for partially hydrogenated oils.<br>2. Choose Heart-Healthy Fats:<br>• Opt for unsaturated fats like those found in avocados, nuts, seeds, and olive oil. These can help improve cholesterol levels.<br>3. Increase Fiber Intake:<br>• Focus on whole grains, fruits, vegetables, legumes, and nuts. Soluble fiber found in oats, barley, beans, lentils, and certain fruits can help lower LDL cholesterol.<br>4. Eat Fatty Fish:<br>• Include fatty fish like salmon, mackerel, and trout in your diet. They're rich in omega-3 fatty acids, which can be beneficial for heart health.<br>5. Limit Dietary Cholesterol:	• Avoid dietary trans fats [ESC].<br>• Reduce dietary saturated fats [ESC].<br>• Increase dietary fibre [ESC].<br>• Consume a dietary pattern that emphasizes intake of vegetables, fruits, whole grains, legumes, healthy protein sources (low-fat dairy products, low-fat poultry (without the skin), fish/seafood, and nuts), and nontropical vegetable oils [AHA-ACC].<br>• Reduce dietary cholesterol [ESC].<br>• Limit intake of sweets, sugar-sweetened beverages, and red meats [AHA-ACC].<br>• Use functional foods enriched with phytosterols [ESC].<br>• Reduce excessive body weight [ESC].<br>• Caloric intake should be adjusted to avoid weight gain, or in overweight/obese patients, to promote weight loss [AHA-ACC].<br>• Increase habitual physical activity [ESC]. In general, adults should be advised to engage in aerobic physical activity 3-4 sessions per week, lasting on average 40 minutes per session and involving moderate-to vigorous-intensity physical activity [AHA-ACC].<br>• Dietary pattern should be adjusted to appropriate calorie requirements, personal and cultural food preferences, and nutritional therapy for other medical conditions including diabetes [AHA-ACC].<br>• Use red yeast rice nutraceuticals [ESC].

<table>
<tr><td>• While dietary cholesterol from foods like eggs and shellfish doesn't have as significant an impact on cholesterol levels as saturated and trans fats, it's still recommended to consume them in moderation.
6. Watch Portion Sizes:
• Be mindful of portion sizes to help maintain a healthy weight, which can contribute to better cholesterol levels.
7. Limit Added Sugars and Refined Carbohydrates:
• Reduce consumption of sugary beverages, sweets, and highly processed foods.
8. Choose Lean Proteins:
• Opt for lean sources of protein like poultry, lean cuts of meat, fish, legumes, and plant-based protein sources like tofu and tempeh.
9. Incorporate Plant Sterols or Stanols:
• These naturally occurring compounds, found in small amounts in plant-based foods, can help lower LDL cholesterol. They are often added to certain margarines and other fortified foods.
10. Stay Hydrated:
• Drink plenty of water, as staying hydrated is important for overall health.
Remember, it's crucial to work with a healthcare professional to develop a comprehensive plan for managing hypercholesterolemia. They can monitor your progress, adjust recommendations as needed, and ensure that your diet is aligned with your overall health goals and any other medical conditions you may have.</td><td></td></tr>
<tr><td colspan="2">Hypertriglyceridemia</td></tr>
<tr><td>I can provide general dietary guidelines that may be helpful for managing hypertriglyceridemia, but it's important to consult a healthcare professional or registered dietitian for personalized advice tailored to your specific situation.
Hypertriglyceridemia is a condition characterized by high levels of triglycerides in the blood. It is often associated with other conditions like obesity, insulin resistance, and metabolic syndrome. Here are some dietary recommendations for managing hypertriglyceridemia:
• Limit Sugar and Refined Carbohydrates:
Reduce your intake of sugary foods and beverages, as well as foods high in refined carbohydrates like white bread, pasta, and pastries. These can contribute to elevated triglyceride levels.
•Focus on Complex Carbohydrates:
Opt for complex carbohydrates like whole grains (e.g., whole wheat bread, brown rice, quinoa, oats) which have a lower impact on blood sugar and triglyceride</td><td>• Reduce total amount of dietary carbohydrates [ESC].
• Reduce intake of mono- and disaccharides [ESC].
• Replace saturated fats with mono- or polyunsaturated fats [ESC].
• Use supplements of n-3 polyunsaturated fats [ESC].
• Reduce alcohol intake [ESC].
• Reduce excessive body weight [ESC].
• Increase habitual physical activity [ESC].</td></tr>
</table>

levels.

- Increase Fiber Intake:

Fiber-rich foods like fruits, vegetables, legumes, and whole grains can help lower triglycerides. Aim for at least 25-30 grams of fiber per day.

- Choose Healthy Fats:

Include sources of healthy fats like avocados, nuts, seeds, and olive oil. These provide essential fatty acids and can help improve lipid profiles.

- Limit Saturated and Trans Fats:

Reduce consumption of foods high in saturated and trans fats, such as fatty cuts of meat, full-fat dairy products, fried foods, and commercially baked goods.

- Increase Omega-3 Fatty Acids:

Fatty fish like salmon, mackerel, sardines, and trout are rich in omega-3 fatty acids, which can help lower triglyceride levels. Consider incorporating these into your diet.

- Limit Alcohol Consumption:

Alcohol can raise triglyceride levels, so it's advisable to limit or avoid alcohol intake.

- Monitor Portion Sizes:

Pay attention to portion sizes to avoid overeating, which can contribute to elevated triglycerides.

- Stay Hydrated:

Drink plenty of water throughout the day to support overall health and metabolism.

- Avoid Fast Food and Processed Foods:

These often contain high levels of unhealthy fats, sugars, and additives, which can contribute to elevated triglycerides.

- Consider Low Glycemic Index Foods:

Low glycemic index foods release sugar slowly into the bloodstream, which can help regulate blood sugar levels and may be beneficial for managing hypertriglyceridemia.

- Consult a Healthcare Professional:

it's crucial to work with a healthcare provider or registered dietitian who can provide personalized recommendations based on your specific health needs and goals.

Remember, these are general guidelines and may not be suitable for everyone. It's important to consult with a healthcare professional who can provide personalized advice based on your individual health status, preferences, and any specific dietary restrictions or conditions you may have.

Type 2 diabetes mellitus	
A well-balanced diet is crucial for managing type 2 diabetes. It can help regulate blood sugar levels, maintain a healthy weight, and reduce the risk of complications. Here are some general dietary guidelines for individuals with type 2 diabetes:<br>1. Focus on Complex Carbohydrates:<br>• Choose whole grains like brown rice, whole wheat bread, quinoa, and oats. These have a lower glycemic index and provide a slower release of glucose into the bloodstream.<br>2. Control Portion Sizes:<br>• Be mindful of portion sizes to avoid overeating, which can cause blood sugar spikes. Use smaller plates, bowls, and utensils to help with portion control.<br>3. Limit Added Sugars and Refined Carbohydrates:<br>• Avoid sugary drinks, candies, and desserts. Be cautious with foods that have hidden sugars, like some sauces, condiments, and processed snacks.<br>4. Incorporate Lean Proteins:<br>• Include sources of lean protein like poultry, fish, tofu, legumes, and low-fat dairy. Protein can help stabilize blood sugar levels.<br>5. Healthy Fats:<br>• Choose sources of healthy fats like avocados, nuts, seeds, and olive oil. Limit saturated and trans fats found in fried foods, fatty cuts of meat, and processed snacks.<br>6. Fiber-Rich Foods:<br>• Include plenty of fruits and vegetables, as they are high in fiber. Fiber helps regulate blood sugar levels and supports overall digestive health.<br>7. Balanced Meals:<br>• Aim for balanced meals that include a combination of carbohydrates, proteins, and healthy fats. This can help maintain steady blood sugar levels throughout the day.<br>8. Limit Processed Foods:<br>• Processed foods often contain hidden sugars, unhealthy fats, and high levels of sodium. Opt for whole, minimally processed foods whenever possible.<br>9. Stay Hydrated:<br>• Drink plenty of water throughout the day. Avoid sugary drinks, as they can lead to rapid spikes in blood sugar levels.<br>10. Monitor Blood Sugar Levels:<br>• Regularly monitor your blood sugar levels to understand how different foods affect you. This can help you make more informed dietary choices.	• Reducing overall carbohydrate intake for individuals with diabetes has demonstrated the most evidence for improving glycemia and may be applied to a variety of eating patterns that meet individual needs and preferences [ADA].<br>• A wide range of carbohydrate intakes are acceptable [EASD]. Carbohydrate intake should emphasize nutrient-dense carbohydrate sources that are high in fiber and minimally processed.<br>• Diets with a low glycaemic index or low glycaemic load can be recommended [EASD].<br>• Intakes of free or added sugars should be below 10% of total energy intake [EASD].<br>• People with diabetes who have overweight or obesity should be supported with evidence-based treatments to achieve and maintain weight loss [EASD] and a minimum weight loss of 5% is recommended [ADA].<br>• There is no ideal macronutrient pattern for people with diabetes [ADA]. A variety of weight-loss diet types and macronutrient compositions can be used for weight-loss induction and maintenance [EASD]; meal plans should be individualized [ADA].<br>• Eating plans should emphasize sources of dietary fibre as nonstarchy vegetables, fruits, legumes, and minimally processed whole grains, as well as dairy products, with minimal added sugars. [EASD, ADA]. Foods naturally high in dietary fibre should be encouraged. Dietary fibre intake should be at least 35 g per day (4 g per 1000 kJ) [EASD] at least 14 g fiber per 1,000 kcal [ADA].<br>• Dietary fats should mainly come from plant-based foods high in both mono- and polyunsaturated fats, such as nuts, seeds and non-hydrogenated non-tropical vegetable oils [EASD].<br>• Saturated- and trans-fat intakes should comprise <10% e <1% respectively [EASD].<br>• Eating foods rich in long-chain n-3 fatty acids, such as fatty fish (EPA and DHA) and nuts and seeds (ALA), is recommended to prevent or treat cardiovascular disease [ADA].<br>• An eating plan emphasizing elements of a Mediterranean eating pattern rich in monounsaturated and polyunsaturated fats may be considered to improve glucose metabolism and lower cardiovascular disease risk [ADA].<br>• Adults with diabetes who drink alcohol should do so in moderation (no more than one drink per day for adult women and no more than two drinks per day for adult men) [ADA].<br>• Sodium consumption should be limited to <2,300 mg/day [ADA].<br>• Non-nutritive sweeteners (NNS) can be used to replace sugars in foods and beverages. [EASD, ADA].

11. Spread Meals Throughout the Day:<br>• Instead of three large meals, consider having smaller, balanced meals and snacks throughout the day. This can help prevent large fluctuations in blood sugar levels.<br>12. Limit Alcohol Consumption:<br>• If you choose to consume alcohol, do so in moderation. Be aware that alcohol can affect blood sugar levels, so monitor them closely.<br>13. Consult with a Registered Dietitian:<br>• A registered dietitian can provide personalized advice based on your specific needs, preferences, and lifestyle.<br>14. Physical Activity: Combine a balanced diet with regular physical activity, as exercise can help improve insulin sensitivity and blood sugar control.<br><br>Remember, it's important to work closely with your healthcare team, including your doctor and a registered dietitian, to create a customized diet plan that suits your individual needs and helps you manage your type 2 diabetes effectively.	
Chronic kidney disease	
I can provide some general guidelines for a diet that may be suitable for someone with chronic kidney disease (CKD), but please keep in mind that individual dietary recommendations should be tailored to each person's specific medical condition, stage of CKD, and other factors. It's important for individuals with CKD to work closely with their healthcare team, including a registered dietitian or nutritionist who specializes in kidney disease, to develop a personalized nutrition plan.<br>Here are some general dietary recommendations for people with chronic kidney disease:<br>1. Limit Protein Intake: Depending on the stage of CKD, protein intake may need to be restricted. This is because the kidneys may have difficulty processing the waste products that come from protein metabolism. High-quality sources of protein like lean meats, poultry, fish, and dairy should be prioritized.<br>2. Control Phosphorus: In advanced stages of CKD, the kidneys may have difficulty regulating phosphorus levels, leading to high levels in the blood. This can be managed by avoiding high-phosphorus foods like dairy products, nuts, seeds, and certain processed foods.<br>3. Monitor Sodium (Salt) Intake: Controlling sodium intake can help manage blood pressure and fluid balance. This may involve limiting processed foods, canned soups, and using herbs and spices for flavor instead of salt.	•Protein intake:<br>- maintain a protein intake of 0.8 g/kg/day in adults with CKD G3–G5. Do not restrict protein intake in adults with sarcopenia, cachexia, or conditions that result in undernutrition [KDIGO].<br>- In adults with CKD 3-5 who are metabolically stable, we recommend, under close clinical supervision, protein restriction with or without keto acid analogs, to reduce risk for end-stage kidney disease (ESKD)/death and improve quality of life (QoL) [KDIGO-KDOQI].<br>• Energy Intake: In adults with CKD 1-5D or posttransplantation who are metabolically stable, it is recommended an energy intake of 25-35 kcal/kg body weight per day based on age, sex, level of physical activity, body composition, weight status goals, CKD stage, and concurrent illness or presence of inflammation to maintain normal nutritional status [KDOQI].<br>• In adults with CKD 1-5D or posttransplantation, it is suggested prescribing folate, vitamin B12, and/or B-complex supplement to correct for folate or vitamin B12 deficiency/insufficiency based on clinical signs and symptoms [KDOQI].<br>• In adults with CKD 1-5D or posttransplantation who are at risk of vitamin C deficiency, it is reasonable to consider supplementation to meet the recommended intake of at least 90 mg/d for men and 75 mg/d for women [KDOQI].<br>•In adults with CKD 1-5D, we suggest to not routinely supplement selenium or zinc since

4. Limit Potassium: In later stages of CKD, potassium levels may need to be controlled. High-potassium foods like bananas, oranges, tomatoes, and potatoes may need to be limited.<br>5. Manage Fluid Intake: Depending on the stage of CKD, fluid intake may need to be restricted to prevent fluid buildup in the body. This can involve monitoring fluid intake from both food and beverages.<br>6. Monitor Blood Sugar Levels: For individuals with diabetes and CKD, it's important to closely manage blood sugar levels through diet and, if necessary, medication.<br>7. Choose Heart-Healthy Fats: Opt for sources of unsaturated fats like avocados, nuts, seeds, and olive oil. Limit saturated and trans fats found in fried foods, fatty cuts of meat, and processed snacks.<br>8. Consider Caloric Intake: Depending on the individual's overall health, energy needs may vary. Some people with CKD may need to adjust their calorie intake to maintain a healthy weight.<br>9. Supplement as Necessary: Depending on blood test results, some individuals with CKD may need specific vitamin or mineral supplements (e.g., vitamin D, iron, B vitamins). This should be determined by a healthcare provider.<br>10. Individualize Diet Plan: Each person's dietary needs and restrictions may vary, so it's important to work with a registered dietitian or nutritionist who specializes in kidney disease to develop a personalized nutrition plan.<br>Remember, these are general guidelines and should not be considered a substitute for personalized advice from a healthcare professional. Always consult with your healthcare team before making significant changes to your diet, especially if you have a medical condition like chronic kidney disease.	there is little evidence that it improves nutritional, inflammatory, or micronutrient status. [KDOQI].<br>• In adults with CKD 1-5D or posttransplantation, it is reasonable when making decisions about phosphorus restriction treatment to consider the bioavailability of phosphorus sources (eg, animal, vegetable, additives) [KDOQI].<br>• In adults with CKD 3-5D or posttransplantation, it is reasonable to adjust dietary potassium intake to maintain serum potassium within the normal range [KDOQI]. Provide advice to limit the intake of foods rich in bioavailable potassium (e.g., processed foods) for people with CKD G3–G5 who have a history of hyperkalemia or as a prevention strategy during disease periods in which hyperkalemia risk may be a concern [KDIGO].<br>• In adults with CKD 3-5, CKD 5D, or posttransplantation, it is recommended a sodium intake to less than 100 mmol/d (or <2.3 g/d) [KDOQI] or <2 g of sodium per day (or <90 mmol of sodium per day, or <5 g of sodium chloride per day) [KDIGO] to reduce blood pressure and improve volume control [KDOQI]. Assessment and education through a registered dietitian or accredited nutrition providers is advised [KDIGO].<br>• Use registered dieticians or accredited nutrition providers to provide information for people with CKD about dietary adaptations regarding sodium, phosphorus, potassium, and protein intake, tailored to their individual needs, and severity of CKD and other comorbid conditions, where available [KDIGO].<br>• In adults with CKD 1-5 not on dialysis or posttransplantation, consider a plant-based "Mediterranean-style" diet in addition to lipid-modifying therapy to reduce cardiovascular risk. [KDOQI-KDIGO].<br>• Advise people with CKD to adopt healthy and diverse diets with a higher consumption of plant-based foods compared to animal-based foods and a lower consumption of ultra-processed foods. [KDIGO]. In adults with CKD 1-4, we suggest that prescribing increased fruit and vegetable intake may decrease body weight, blood pressure, and net acid production [KDOQI].<br>• Encourage people with CKD to undertake physical activity compatible with cardiovascular health, tolerance, and level of frailty; achieve an optimal body mass index (BMI); and not use tobacco products. Referral to providers and programs (e.g. psychologists, dieticians, physical and occupational therapy, and smoking cessation programs) should be offered where indicated and available [KDIGO].
Obesity	
Dietary recommendations for obesity generally focus on achieving a sustainable and balanced caloric intake while promoting nutrient-dense foods. It's important	• Calorie restriction should be individualised and take account of nutritional habits, physical activity, co-morbidities and previous dieting attempts [EASO].

to consult with a healthcare professional or registered dietitian before making significant changes to your diet, especially if you have specific health concerns or conditions. Here are some general dietary recommendations for managing obesity:<br>• Caloric Deficit: To lose weight, you generally need to consume fewer calories than you expend. This can be achieved through a combination of reducing calorie intake and increasing physical activity.<br>• Balanced Diet: Aim for a balanced diet that includes a variety of whole foods, such as fruits, vegetables, whole grains, lean proteins, and healthy fats. This provides a range of nutrients essential for overall health.<br>• Portion Control: Be mindful of portion sizes to avoid overeating. Pay attention to hunger and fullness cues, and try to stop eating when you're satisfied, not overly full.<br>• Limit Processed Foods and Sugars: Reduce consumption of highly processed foods, sugary snacks, and sugary beverages. These often contain excess calories, added sugars, and low nutritional value.<br>• Limit Saturated and Trans Fats: Reduce intake of foods high in saturated and trans fats, such as fried foods, processed snacks, and fatty cuts of meat. Opt for healthier fats like those found in avocados, nuts, and olive oil.<br>• Increase Fiber Intake: Fiber-rich foods like fruits, vegetables, whole grains, and legumes can help you feel full and satisfied, which can be helpful for weight management.<br>• Hydration: Drink plenty of water throughout the day. Sometimes thirst can be mistaken for hunger, so staying well-hydrated can help manage appetite.<br>• Regular Meals and Snacks: Eating regular, balanced meals and snacks can help stabilize blood sugar levels and prevent overeating later in the day.<br>• Mindful Eating: Pay attention to what you're eating, savor each bite, and eat without distractions. This can help you recognize when you're full and reduce the tendency to eat out of habit or emotional reasons.<br>• Limit Alcohol: Alcoholic beverages can contribute extra calories without providing much nutritional benefit, so it's advisable to consume them in moderation or avoid them altogether.<br><br>Consult a Professional: If possible, seek guidance from a registered dietitian or healthcare provider who can create a personalized nutrition plan based on your specific needs, preferences, and any underlying health conditions.<br><br>Remember that individual dietary needs can vary based on factors like age,	• Balanced hypocaloric diets result in clinically meaningful weight loss regardless of which macronutrients they emphasise [EASO].<br>• Nutrition recommendations for adults of all body sizes should be personalized to meet individual values, preferences and treatment goals to support a dietary approach that is safe, effective, nutritionally adequate, culturally acceptable and affordable for long-term adherence [Canadian].<br>• Balanced hypocaloric diets can be tailored to individual patients on the basis of their personal and cultural preferences and may therefore have the best chance for long-term success (e.g. Mediterranean diet) [EASO].<br>• Dietary advice should encourage healthy eating and emphasise the need to increase consumption of vegetables, beans, legumes, lentils, grain, unsweetened cereals and fibre, and to substitute low-fat dairy products and meats for high-fat alternatives [EASO].<br>• Decrease the size of food portions [EASO].<br>• Pulses, vegetables, fruit, whole grains, dairy foods are reccomended [Canadian CPG].<br>• It is recommended to avoid foods containing added sugars and solid fats, as well as consumption of sugary drinks and alcohol-containing beverages [EASO].<br>• Avoid snacking between meals – Do not skip breakfast and avoid eating in the nighttime [EASO].<br>• Decrease energy density of foods and drinks [EASO].<br>• It should also emphasise increased intake of seafood [EASO].

gender, activity level, and any underlying health conditions, so it's important to tailor recommendations to your specific situation. Additionally, sustainable changes to your eating habits are more likely to lead to long-term success in managing obesity.	
Arterial hypertension	
A balanced and heart-healthy diet can play a significant role in managing hypertension (high blood pressure). Here are some dietary recommendations for individuals with hypertension:<br>1.Limit Sodium Intake:<br>•Aim for less than 2,300 milligrams of sodium per day. Ideally, try to reduce it further to 1,500 milligrams, especially if you have more severe hypertension or are at higher risk for heart disease.<br>2.Emphasize Whole Foods:<br>•Consume a diet rich in fruits, vegetables, whole grains, lean proteins, and healthy fats. These foods are naturally lower in sodium and high in essential nutrients.<br>3.Potassium-Rich Foods:<br>•Increase your intake of potassium, which can help counterbalance the effects of sodium. Foods high in potassium include bananas, oranges, potatoes, spinach, and beans.<br>4.Reduce Processed Foods:<br>•Processed foods are often high in sodium. This includes canned soups, processed meats, salty snacks, and most restaurant/fast food.<br>5.Limit Added Sugars and Refined Carbohydrates:<br>•These can contribute to weight gain and potentially worsen blood pressure. Choose whole, unprocessed foods whenever possible.<br>6.Moderate Alcohol Intake:<br>•If you consume alcohol, do so in moderation. For men, this typically means up to two drinks per day, and for women, one drink per day.<br>7.Limit Caffeine:<br>•While moderate caffeine intake is generally fine for most people, some individuals are more sensitive to its effects on blood pressure. Monitor how caffeine affects you personally.<br>8.Choose Lean Proteins:<br>•Opt for lean sources of protein like poultry, fish, legumes, and tofu. Limit red meat consumption, and choose lean cuts when you do eat it.<br>9.Healthy Fats:<br>•Replace saturated and trans fats with healthier fats like monounsaturated and	• Salt reduction: reduce salt added when preparing foods, and at the table. Avoid or limit consumption of high salt foods such as soy sauce, fast foods and processed food including breads and cereals high in salt [ESH-ISH].<br>• Eating a diet that is rich in whole grains, fruits, vegetables, polyunsaturated fats and dairy products and reducing food high in sugar, saturated fat and trans fats [ISH].<br>• Increase intake of vegetables high in nitrates known to reduce BP, such as leafy vegetables and beetroot [ISH]. Other beneficial foods and nutrients include those high in magnesium, calcium [ISH], and potassium [ISH-ESH].<br>• Moderation of alcohol consumption [ESH-ISH]: the recommended daily limit for alcohol consumptions is 2 standard drinks for men and 1.5 for women (10 g alcohol/standard drink). Avoid binge drinking [ISH].<br>• Moderate consumption of coffee, green and black tea [ESH-ISH].<br>• Regular physical activity [ESH-ISH]: moderate intensity aerobic exercise (walking, jogging, cycling, yoga, or swimming) for 30 minutes on 5–7 days per week or HIIT (high intensity interval training) which involves alternating short bursts of intense activity with subsequent recovery periods of lighter activity. Strength training also can help reduce blood pressure. Performance of resistance/strength exercises on 2–3 days per week [ISH].<br>• Weight reduction [ESH-ISH].<br>• DASH diet [ESH-ISH].

polyunsaturated fats. These are found in foods like avocados, nuts, seeds, and olive oil.<br>10.Watch Portion Sizes:<br>•Be mindful of portion sizes to help control calorie intake, which can impact your overall health.<br>11.Stay Hydrated:<br>•Drink plenty of water. Proper hydration is important for overall health, including cardiovascular health.<br>12.Consider the DASH Diet:<br>•The Dietary Approaches to Stop Hypertension (DASH) diet is a well-researched approach specifically designed to lower blood pressure. It emphasizes fruits, vegetables, whole grains, and lean proteins while reducing sodium intake.<br>Remember, it's always best to consult with a healthcare professional or a registered dietitian before making significant changes to your diet, especially if you have a medical condition like hypertension. They can provide personalized advice based on your specific health situation.	
Sarcopenia	
Sarcopenia is a condition characterized by the loss of muscle mass and function, often associated with aging. Proper nutrition plays a crucial role in managing and preventing sarcopenia. Here are some dietary recommendations for individuals dealing with sarcopenia:<br>•Adequate Protein Intake: Protein is essential for muscle maintenance and repair. Aim for a higher intake of high-quality protein sources like lean meats, poultry, fish, eggs, dairy products, legumes, and plant-based protein options like tofu and tempeh.<br>• Distribute Protein Intake: It's beneficial to spread protein intake evenly throughout the day rather than consuming most of it in one meal. This helps provide a steady supply of amino acids for muscle repair and maintenance.<br>•Resistance Exercise: Combining proper nutrition with regular resistance exercise (strength training) is crucial for preserving and building muscle mass. This stimulates muscle protein synthesis and helps counteract the effects of sarcopenia.<br>•Adequate Caloric Intake: Ensure you are consuming enough calories to support your activity levels and maintain muscle mass. However, it's important to strike a balance and not overconsume calories, as excessive body fat can also negatively impact muscle health.<br>•Micronutrient-Rich Diet: Make sure your diet includes a variety of fruits and vegetables to provide essential vitamins and minerals. Vitamins like vitamin D,	• Diet should include at least 1.0-1.2 g protein/kg body weight/day. For certain older adults who have acute or chronic illnesses, 1.2-1.5 g protein/kg body weight/day may be indicated, with even higher intake for individuals with severe illness or injury [ESPEN].<br>• Consider protein supplementation/a protein-rich diet for older adults with sarcopenia [ICFSR].<br>• In patients with sarcopenia, prescription of resistance-based training may be effective to improve lean mass, strength and physical function [ICFSR- ESPEN].<br>• Nutritional intervention should be combined with a physical activity intervention [ICFSR].<br>• Clinicians may also consider discussing with patients the importance of adequate calorie and protein intake [ICFSR].<br>• Insufficient evidence exists to determine whether a Vitamin D supplementation regime by itself is effective in older adults with sarcopenia [ICFSR].

calcium, and vitamin B12, along with minerals like calcium and magnesium, are important for muscle health.<br>•Omega-3 Fatty Acids: Omega-3 fatty acids, found in fatty fish like salmon, mackerel, and sardines, have anti-inflammatory properties that can support muscle health.<br>•Limit Processed Foods and Added Sugars: Minimize the consumption of processed foods, sugary snacks, and sugary drinks, as they can contribute to inflammation and may negatively impact muscle health.<br>•Hydration: Staying properly hydrated is important for overall health, including muscle function. Water helps transport nutrients to your cells and remove waste products.<br>•Consider Supplements: In some cases, especially if there are deficiencies, supplements may be recommended. This should be done under the guidance of a healthcare professional.<br>Remember, it's important to approach any dietary changes or supplements with guidance from a healthcare provider, especially if you have any underlying health conditions or are taking medications. They can provide personalized recommendations based on your individual circumstances.	
NAFLD	
Non-Alcoholic Fatty Liver Disease (NAFLD) is a condition where excess fat accumulates in the liver, not caused by alcohol consumption. It's essential to manage NAFLD through a balanced diet and lifestyle changes. Here are some dietary recommendations for NAFLD:<br>1. Reduce Saturated and Trans Fats:<br>• Limit your intake of saturated fats found in red meat, processed foods, and full-fat dairy products.<br>• Avoid trans fats found in partially hydrogenated oils and many processed foods.<br>2. Emphasize Healthy Fats:<br>• Include sources of healthy fats like avocados, nuts, seeds, and olive oil.<br>3. Increase Fiber Intake:<br>• Consume plenty of fiber-rich foods like fruits, vegetables, whole grains, and legumes. Fiber helps in digestion and can help regulate blood sugar levels.<br>4. Choose Lean Proteins:<br>• Opt for lean sources of protein like poultry, fish, beans, and tofu. Limit red meat consumption.<br>5. Control Portion Sizes:<br>• Be mindful of portion sizes to avoid overeating, which can contribute to weight gain.	• Patients with NAFLD who are overweight or obese should follow a diet with a caloric deficit leading to weight loss [ESPEN, AASLD]<br>• In order to achieve weight loss, a hypocaloric diet shall be followed according to current obesity guidelines irrespective of the macronutrient composition [ESPEN]<br>• When possible, diets with limited carbohydrates and saturated fat and enriched with high fiber and unsaturated fats [AASLD, ESPEN]<br>• Mediterranean diet should be encouraged due to their additional cardiovascular benefits [AASLD].<br>• A Mediterranean diet (MedD) should be advised to improve steatosis and insulin sensitivity [ESPEN].<br>• NAFLD patients shall be encouraged to abstain from alcohol in order reduce risk for comorbidity and to improve liver biochemistry and histology [ESPEN].<br>• Until further data regarding their efficacy are available, 3-fatty acids and antioxidants (e.g. vitamin C, resveratrol, anthocyanin, bayberries) cannot be recommended to treat NAFLD [ESPEN].<br>• Patients with NAFLD should be strongly encouraged to increase their activity level to the extent possible. Individualized prescriptive exercise recommendations may increase sustainability and have benefits independent of weight loss [AASLD].<br>• Coffee consumption (caffeinated or not) of at least 3 cups daily is associated with

6. Limit Added Sugars:<br>• Minimize the consumption of sugary foods and beverages, as excessive sugar intake can lead to weight gain and insulin resistance.<br>7. Limit Sugar and High-Fructose Corn Syrup: Avoid or limit foods and beverages high in added sugars, such as sugary drinks, candies, and desserts. This includes avoiding high-fructose corn syrup, which is commonly found in many processed foods.<br>8. Moderate Carbohydrate Intake:<br>• Choose complex carbohydrates like whole grains, fruits, and vegetables over refined grains.<br>9. Watch Your Sodium Intake:<br>• Reducing sodium can help manage fluid retention and blood pressure. Avoid excessive salt intake from processed foods.<br>10. Stay Hydrated:<br>• Drink plenty of water throughout the day. Avoid sugary beverages and excessive caffeine.<br>11. Include Foods Rich in Antioxidants:<br>• Berries, leafy greens, nuts, and seeds are rich in antioxidants, which can help protect liver cells.<br>12. Exercise Regularly:<br>• Incorporate regular physical activity into your routine. Exercise can help improve insulin sensitivity, reduce fat accumulation, and support overall liver health.<br>13. Limit Caffeine and Alcohol:<br>• While moderate coffee consumption may have some benefits for the liver, it's important to limit caffeine intake from other sources. Alcohol should be avoided or consumed in moderation, as it can exacerbate liver damage.<br>14. Consider Supplements (Under Medical Supervision):<br>• Some supplements like vitamin E, omega-3 fatty acids, and certain antioxidants may be beneficial, but it's important to consult a healthcare provider before adding any supplements to your diet.<br>15. Consult a Registered Dietitian:<br>• Consider working with a registered dietitian who can provide personalized dietary recommendations based on your specific health needs and goals.<br>Remember, it's crucial to consult with a healthcare professional or a registered dietitian before making significant changes to your diet, especially if you have a medical condition like NAFLD. They can provide you with personalized advice and monitor your progress.	less advanced liver disease [AASLD].<br>• Excessive fructose consumption in particular increases the risk of NAFLD, NASH, and advanced fibrosis independent of calorie intake [AASLD].
