# Supplementary material for: Is ChatGPT an Effective Tool for Providing Dietary Advice?
Source: Nutrients. 2024 Feb 6;16(4):469. doi: 10.3390/nu16040469 (PMC10892804; doi:10.3390/nu16040469)
Supplement: Supplementary file 1 [file nutrients-16-00469-s001.zip › Table S2.pdf]

Table S2. Inappropriate, unsupported, and missing ddvice from ChatGPT responses

	Inappropriate	Not supported	Missing
Dyslipidemia			• Dietary pattern should be adjusted to appropriate calorie requirements, personal and cultural food preferences, and nutritional therapy for other medical conditions including diabetes [AHA-ACC].<br>• Use red yeast rice nutraceuticals [ESC].
Arterial hypertension			• Regular physical activity [ESH-ISH]: moderate intensity aerobic exercise (walking, jogging, cycling, yoga, or swimming) for 30 minutes on 5–7 days per week or HIIT (high intensity interval training) which involves alternating short bursts of intense activity with subsequent recovery periods of lighter activity. Strength training also can help reduce blood pressure. Performance of resistance/strength exercises on 2–3 days per week [ISH].
Obesity	Regular Meals and Snacks: Eating regular, balanced meals and snacks can help stabilize blood sugar levels and prevent overeating later in the day.		• Decrease energy density of foods and drinks [EASO].<br>• It should also emphasize increased intake of seafood [EASO].
Type 2 diabetes mellitus		Spread Meals Throughout the Day: Instead of three large meals, consider having smaller, balanced meals and snacks throughout the day. This can help prevent large fluctuations in blood sugar levels.	• There is no ideal macronutrient pattern for people with diabetes [ADA]. A variety of weight-loss diet types and macronutrient compositions can be used for weight-loss induction and maintenance [EASD]; meal plans should be individualized [ADA].<br>• Sodium consumption should be limited to <2,300 mg/day [ADA].<br>• Non-nutritive sweeteners (NNS) can be used to replace sugars in foods and beverages. [EASD, ADA].
NAFLD	Consider Supplements (Under Medical Supervision):<br>Some supplements like vitamin E, omega-3 fatty acids, and certain antioxidants may be beneficial, but it's important to consult a healthcare provider before adding any supplements to your diet.		• In order to achieve weight loss, a hypocaloric diet shall be followed according to current obesity guidelines irrespective of the macronutrient composition [ESPEN]<br>• Mediterranean diet should be encouraged due to their additional cardiovascular benefits [AASLD].A Mediterranean diet (MedD) should be advised to improve steatosis and insulin sensitivity [ESPEN]
Chronic kidney disease			In adults with CKD 1-5 not on dialysis or posttransplantation, consider a plant-based “Mediterranean-style” diet in addition to lipid-modifying therapy to reduce cardiovascular risk. [KDOQI-KDIGO].<br>• Advise people with CKD to adopt healthy and diverse diets with a higher consumption of plant-based foods compared to animal-based foods and a lower consumption of ultra-processed foods. [KDIGO]. In adults with CKD 1-4, we suggest that prescribing increased fruit and vegetable intake may decrease body weight, blood pressure, and net acid production [KDOQI].
Sarcopenia			
